# Effect of a Brief Outreach Educational Intervention on the Translation of Acute Poisoning Treatment Guidelines to Practice in Rural Sri Lankan Hospitals: A Cluster Randomized Controlled Trial

**DOI:** 10.1371/journal.pone.0071787

**Published:** 2013-08-19

**Authors:** Lalith Senarathna, Nick A. Buckley, Michael J. Dibley, Patrick J. Kelly, Shaluka F. Jayamanna, Indika B. Gawarammana, Andrew H. Dawson

**Affiliations:** 1 South Asian Clinical Toxicology Research Collaboration, Faculty of Medicine, University of Peradeniya, Peradeniya, Sri Lanka; 2 Sydney School of Public Health, University of Sydney, Sydney, NSW, Australia; 3 Professorial Medicine Unit, POW Clinical School, University of New South Wales, Sydney, Australia; 4 Department of Medicine, Faculty of Medicine, University of Kelaniya, Kelaniya, Sri Lanka; 5 Department of Medicine, Faculty of Medicine, University of Peradeniya, Peradeniya, Sri Lanka; 6 Royal Prince Alfred Clinical School, University of Sydney, Sydney, NSW, Australia; Universidade de São Paulo, Brazil

## Abstract

**Background:**

In developing countries, including Sri Lanka, a high proportion of acute poisoning and other medical emergencies are initially treated in rural peripheral hospitals. Patients are then usually transferred to referral hospitals for further treatment. Guidelines are often used to promote better patient care in these emergencies. We conducted a cluster randomized controlled trial (ISRCTN73983810) which aimed to assess the effect of a brief educational outreach (‘academic detailing’) intervention to promote the utilization of treatment guidelines for acute poisoning.

**Methods and Findings:**

This cluster RCT was conducted in the North Central Province of Sri Lanka. All peripheral hospitals in the province were randomized to either intervention or control. All hospitals received a copy of the guidelines. The intervention hospitals received a brief out-reach academic detailing workshop which explained poisoning treatment guidelines and guideline promotional items designed to be used in daily care. Data were collected on all patients admitted due to poisoning for 12 months post-intervention in all study hospitals. Information collected included type of poison exposure, initial investigations, treatments and hospital outcome. Patients transferred from peripheral hospitals to referral hospitals had their clinical outcomes recorded. There were 23 intervention and 23 control hospitals. There were no significant differences in the patient characteristics, such as age, gender and the poisons ingested. The intervention hospitals showed a significant improvement in administration of activated charcoal [OR 2.95 (95% CI 1.28–6.80)]. There was no difference between hospitals in use of other decontamination methods.

**Conclusion:**

This study shows that an educational intervention consisting of brief out-reach academic detailing was effective in changing treatment behavior in rural Sri Lankan hospitals. The intervention was only effective for treatments with direct clinician involvement, such as administering activated charcoal. It was not successful for treatments usually administered by non-professional staff such as forced emesis for poisoning.

**Trial Registration:**

Controlled-Trials.com ISRCTN73983810 ISRCTN73983810

## Introduction

Clinical guidelines from governments, professional associations or healthcare organizations are a common feature of clinical practice around the world and facilitate more consistent, effective and efficient medical practice [1], [2]. Clinical guidelines assist decision-making about appropriate care for specific clinical conditions and are important instruments in implementing evidence based medicine [3], [4]. Although clinical guidelines can help practitioners to improve their professional practice, the quality of care, and the subsequent outcomes of their patients, this does not automatically mean that guidelines will be applied following their introduction [3]. The application of clinical guidelines depends on factors such as the organizational/environment characteristics, the clarity and the complexity of the guidelines and the strategies used to encourage use. Hence, it is essential to explore and assess effective strategies to translate guidelines into clinical practice. [4], [5].

Treatment guidelines have been issued for many important health problems in Sri Lanka [6], [7]. Most are freely available and distributed to hospitals mainly by the Ministry of Health and professional associations. Acute poisoning is recognized to be a major health problem in Sri Lanka [8], [9]. Evidence-based poisoning treatment guidelines were developed by the national poison information centre in 1994, the third edition was revised and published in 2007 [10]. Printed copies are distributed to all hospitals in the country and all registered medical officers can receive a copy on request. Despite being widely available, previous research reported that the poisoning treatment guidelines were poorly utilized in rural peripheral hospitals in Sri Lanka [11].

Rural peripheral hospitals play a major role in treating acute poisoning as more than 80% of patients are first admitted to these hospitals [12]. Here they receive initial treatment and are then usually transferred to a secondary care hospital for further treatment. Improved early treatment of these patients in peripheral hospitals may potentially reduce mortality and morbidity of acute poisoning patients [13].

Different approaches have been used to integrate standard clinical guidelines into clinical practice. Previously reported effective educational approaches include: interactive and problem based learning from educational workshops to improve professional competence within a group [14], [15]; behavioral approaches including audit/feedback or monitoring systems with reminders [16]; distance learning approaches [17], [18], and social interaction approaches with outreach visits by opinion leaders, and influential key people in the social network [19], [20], [21]. The success of all these methods depends on the setting or the type of hospital. In Sri Lanka, rural primary care peripheral hospitals are scattered across a wide geographic area and the distance from the main referral hospitals can vary from 8 to 110 km. Currently centralized educational programs are the main method of training and providing continuing education for doctors, nurses and other staff in this setting. However previous qualitative studies have revealed that centralized programs are viewed negatively by peripheral hospital staff [11]. The logistic difficulties in travelling to and from these meetings and the lack of staff to cover leave were the primary reasons given for the negative attitudes, and help explain the low participation in this type of training of health care staff from remote hospitals. Preference was expressed for locally delivered training programs that would provide wider access to staff. The logistics of providing training in local centers suggested that delivering brief interventions would be achievable using existing resources. Hence in this study we adopted the principles and tools of academic detailing [21], [22] to provide a brief out-reach education intervention designed to promote key messages from the poisoning treatments guidelines (Table 1). We then assessed whether this intervention was effective in changing the treatment behavior of rural peripheral hospital staff members.

**Table 1:** Agreement of the intervention with academic detailing principles.

Components of Academic Detailing*	Strategies Used in This Study
Conducting interviews to investigate baseline knowledge/motivations	**Based on the findings from previous studies on the use of poisoning treatment guidelines in the same setting**
Focusing programs on specific staff categories and opinion leaders	**Focused on staff categories who are involved in treating acute poisoning patients**
Defining clear educational and behavioral objectives	**Promoted and explained key messages from the poisoning treatment guidelines aiming to improve treatment practices**
Establishing credibility through a respected organizational identity	**The key messages are from poisoning treatment guidelines from National Poison Centre of Sri Lanka & were delivered by senior and influential consultant physicians with policy support from provincial health authorities**
Stimulating active physician participation in educational interactions	**Workshops were interactive and active participation was encouraged**
Using concise graphic educational materials	**Used graphical wall charts which summarized the key messages**
Highlighting and repeating the essential messages	**Used promotional materials in hospital wards that repeated key messages by promoting the use of guidelines and wall charts**

* (Soumerai SB, Avorn J (1990) Principles of educational outreach (‘academic detailing’) to improve clinical decision making. The Journal of the American Medical Association 263∶549–556).

## Methods

The protocol for this trial and supporting CONSORT checklist are available as supporting information; see Checklist S1 and Protocol S1.

### Ethics Statement

This study protocol was reviewed and approved by the Human Research Ethics Committee of University of Sydney Australia (Ref number 12083) and the Ethics Review Committee of University of Peradeniya, Sri Lanka.

### Study Setting and Design

A cluster randomized controlled trial (ISRCTN73983810) was conducted in partnership with the Provincial Department of Health Services in the North Central Province of Sri Lanka. This clinical trial was registered 5 months after the date of first enrollment because it was not clear at the time we designed the study whether it fulfilled the clinical trial registry requirements. We registered the study after the educational intervention, but there was no change in study protocol and no analysis of the partially collected outcome data.

The North Central Province consists of two districts; Anuradhapura, which has 59 peripheral health care units and Polonnaruwa, which has 22 peripheral health care units. Of those units, only peripheral hospitals with in-patient facilities were considered for this study. There were only 34 peripheral hospitals with in-patient facilities in Anuradhapura district and 12 in Polonnaruwa district. Hence a total of 46 were eligible for this study.

### Randomization

All 46 eligible hospitals were randomly allocated to either intervention or control groups using matched randomization with stratification by district. In each stratum, hospitals were ranked and then ordered based on the number of patient beds and the number of annual admissions to represent the size and patient volume. Adjoining hospitals in the ranked list were paired (a matched pair). The random function in excel was used (by an investigator (NAB) unfamiliar with the hospitals) to select the first or second hospital in each pair to have the intervention with the other hospital then assigned to be a control.

### Intervention

The academic detailing intervention was designed to deliver key messages from the poisoning treatment guidelines [10]: including administering activated charcoal; avoiding forced emesis and risky gastric lavage methods; giving pralidoxime for organophosphate poisoning; and giving methionine (antidote) for paracetamol poisoning.

**Figure 1:**
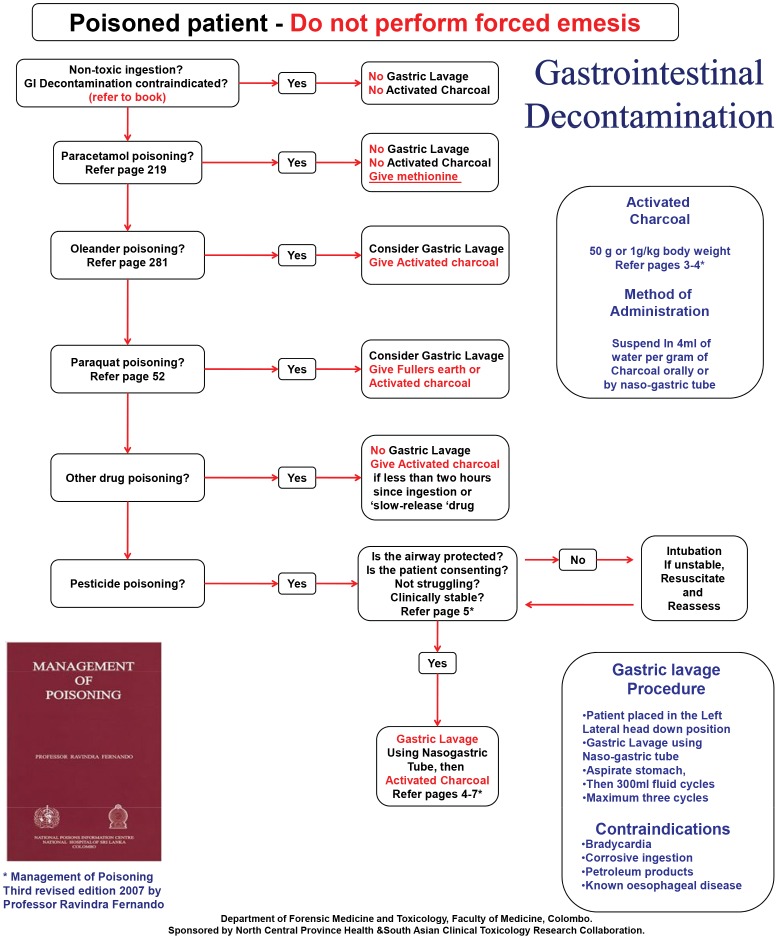
Wall chart to display guidelines on gastric decontamination.

A team that consisted of a locally known senior consultant physician, the principal researcher (LS) and research assistants visited each intervention hospital. The Provincial Department of Health Services scheduled the educational workshop and instructed all staff to attend. The intervention consisted of a presentation from the consultant physician about key messages from the guidelines. A standard set of slides and a graphical wall chart (Figure 1) were used to present the key messages and to facilitate discussion. Following the presentation there was an interactive discussion session with participants based upon questions arising from their experiences. The entire session took approximately 2 to 2.5 hours, At the end of the session, each hospital ward was given copies of the book containing poisoning treatment guidelines [10], wall charts with a graphical summary of the guidelines (Figure 1), cardboard document folders, and notice boards with a promotional message about using guidelines. Each participant was also given a pen with the same message. The number of participating staff was recorded by research assistants. Control hospitals received copies of the poisoning treatment guidelines book but no other components of the intervention. The intervention incorporated many of the proven effective features of the academic detailing (Table 1) [22].

### Data Collection

Intervention and control hospitals were followed up for 12 months post-intervention to collect data at the individual patient level. In Sri Lankan health care system, hospital medical records are kept under the care of the Medical Officer In-Charge of the institution and the Provincial Department of Health Services (PDHS). De-identified data from these records is used in health care planning. In this study, data collectors had PDHS appointments and functioned as part of the PDHS epidemiology audit staff. The PDHS requested all hospitals to keep the poisoned patient records in hospital wards for data extraction (done within 3–4 weeks of admission). Once primary and referral hospital data was linked, data for subsequent analysis was de-identified. The ethics committee and province saw these aspects as part of clinical audit and did not require written consent to be obtained from individual patients. Data was collected on all patients above 12 years of age admitted to a study hospital during this period with a history of acute poisoning. The admission log books in each hospital were also checked to ensure that no patients were missed. The poison product label was usually brought in with the patient and information from it was recorded by peripheral hospital staff in the medical record. A trained research assistant extracted data from the medical record on exposure, clinical assessment, treatment and outcome details using a structured data collection form.

The pre-specified primary outcomes to be assessed within the 12 months post-intervention period in this study were administration of activated charcoal, the use of gastric emptying (forced emesis and gastric lavage), use of pralidoxime in organophosphate poisoning, and use of methionine for paracetamol poisoning. The following were secondary outcomes: the number of deaths from poisoning, the extent of irreversible acetylcholinesterase (AChE) inhibition following organophosphate ingestion, and the estimated cost of treatments (including the cost of transfers to secondary care).

Data about the availability and the level of activated charcoal stocks were also collected. The proportion of hospitals having sufficient stocks of activated charcoal to treat the average number of patients/month (>6 doses) was also compared.

Patients transferred to the referral hospital in one district (Anuradhapura) were examined and interviewed by doctors directly involved in the patients care to record the gastrointestinal decontamination performed in the peripheral hospital. This occurred in the context of an observational cohort study we have been conducting since 2002 which has been reviewed by multiple local ethics review committees (University of Peradeniya, Sri Lanka Medical Association (SLMA), and University of Colombo) and ethics review committee of Australian National University and it was deemed that individual patient consent was not required. Some of the cohort results have been published [23], [24]. This data was compared to the medical records from peripheral hospitals to test the validity of the peripheral hospital record of treatment. (Linking used an algorithm based on hospital name, patient name, age, gender, date/time and poison).

### Sample Size

We anticipated that a study with one year follow up would allow sufficient recruitment to detect clinically meaningful changes in two of our primary outcomes activated charcoal and use of forced emesis. To detect an increase from 35% (historical rate) to 50% in activated charcoal would require 22 hospitals and 990 patients in each group. A reduction from 60% (historical rate) to 40% of patients receiving forced emesis would require 13 hospitals and 585 patients in each group. These calculations were based on a significance level of 0.05, 80% power and assuming an average cluster size of 45 and a within matched-pair intra cluster correlation of 0.1 [25], [26]. Although pralidoxime, methionine were specified primary outcomes, it was not feasible to power the study for these outcomes. The most likely impact of their use would have been on secondary outcomes of acetylcholinesterase inhibition (pralidoxime) and treatment costs (methionine).

### Statistical Analysis

Data analysis was performed on an intention to treat basis, and analyzed at the individual level 6 and 12 months following the intervention. Binary and multinomial outcomes were analyzed using logistic regression and multinomial models respectively. To account for cluster randomization, and pairing of hospitals within districts, each model included a random effect for hospital and a random effect for the matched pairs. As both treatment and outcome is highly dependent upon the type of poisoning and the resources available in the hospital, an analysis was also conducted which adjusted for these factors. Kappa statistics were used to measure agreement between the independent peripheral and referral hospital recording of activated charcoal. Analysis was conducted using STATA® statistical software [27].

## Results

**Figure 2:**
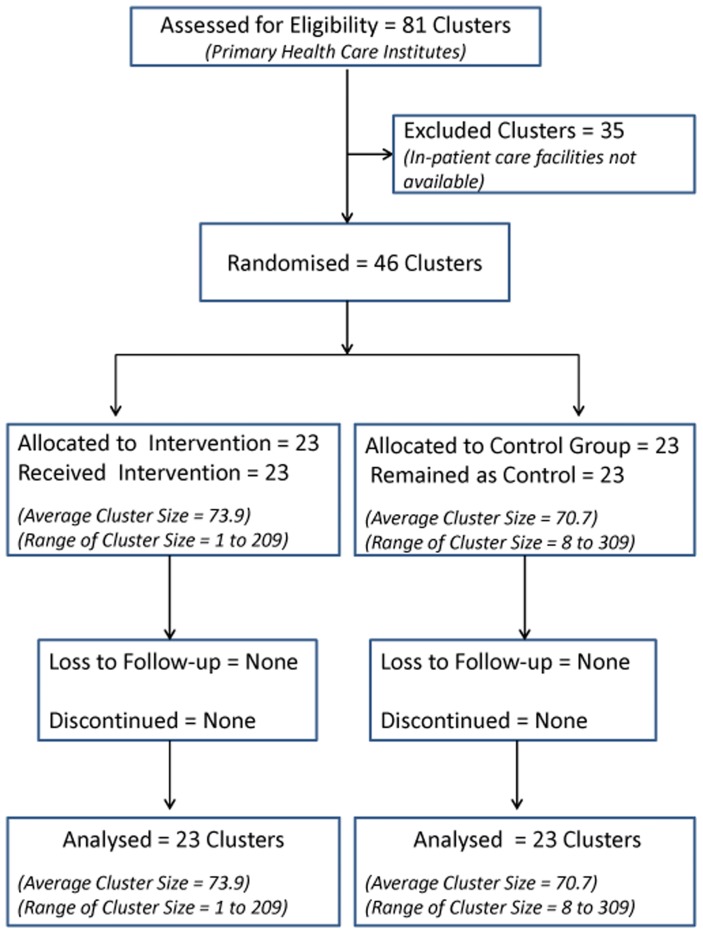
Participant flow chart.

Data from all 46 hospitals (34 from Anuradhapura district and 12 from Polonnaruwa district) were included in the analysis (Figure 2).

There were 3324 acutely poisoned patients admitted to these 46 peripheral hospitals in the year after the intervention – from September 2008 to September 2009 - (1625 in control and 1699 in intervention hospitals). Except for type of hospital, there were no differences in hospital or patient characteristics between the intervention and control groups (Tables 2 and 3).

**Table 2:** Baseline characteristics of study cluster hospitals in North Central Province of Sri Lanka.

	Control		Intervention	
	Hospitals	Admissions	Hospitals	Admissions
	n	n (% )	n	n (%)
**All hospitals by Districts**				
Anuradhapura	17	1075 (66)	17	1193 (70)
Polonnaruwa	6	550 (34)	6	506 (30)
**Hospital Category**				
Base/District Hospitals	5	757 (47)	4	576 (34)
Peripheral Hospitals	6	397 (24)	3	452 (27)
Rural Hospitals	12	471 (29)	16	671 (39)
**No. of Hospital Beds**				
30 or less	8	266 (17)	9	295 (17)
31 to 60	9	468 (29)	7	669 (40)
61 to 100	3	233 (14)	4	358 (21)
101 or more	3	658 (40)	3	377 (22)
**Total**	**23**	**1,625**	**23**	**1,699**

**Table 3:** Baseline characteristics of poisoned patients admitted to study hospitals in North Central Province of Sri Lanka.

	Control	Intervention
	n (%)	n (%)
**Gender**		
Male	824 (51)	819 (48)
Female	801 (49)	880 (52)
**Age Groups**		
12–19	507 (31)	517 (30)
20–29	529 (33)	575 (34)
30–39	239 (15)	280 (17)
40–49	187 (11)	171 (10)
> = 50	163 (10)	156 (9)
**Type of Poison**		
Organophosphates and Carbamates	310 (19)	283 (17)
Paraquat	20 (1)	30 (2)
Other Pesticides	330 (20)	352 (21)
Medicine	289 (18)	328 (19)
Oleander	244 (15)	203 (12)
Hydrocarbon	93 (6)	90 (5)
Other & Unknown Poison	339 (21)	413 (24)
**Total**	**1,625**	**1,699**

### Primary Outcomes

There was an increase in the use of activated charcoal in the intervention hospitals (adjusted odds ratio (aOR): 2.95 (95% CI: 1.28–6.80) ). The aOR was also higher and statistically significant for each type of poison (Table 4). The use of forced emesis was lower in the intervention group, but this was not significant (53% vs 60%, aOR: 0.99 (95% CI 0.79–1.24) (Table 4). The planned sub-group analysis of all primary outcomes 6 months following intervention show similar results to the 12 months analyses (Table S1).

**Table 4:** Primary outcomes with adjusted odds ratios to assess the effect of intervention over the 12 months follow-up period in intervention and control hospitals in North Central Province of Sri Lanka.

	Control	Intervention	Adjusted for matched pairs & clustering,Not adjusted for covariates			Adjusted for matched pairs & clustering, & Adjusted for covariates #		
	Y/n (%)	Y/n (%)	OR	95% CI	P value	OR	95% CI	P value
**Activated Charcoal**								
Overall	555/1625 (34)	649/1699 (38)	**2.29**	(1.04–5.07)	0.04	**2.95**	(1.28–6.80)	0.01
Pesticides	295/660 (45)	319/665 (48)	**2.37**	(0.96–5.85)	0.06	**2.64**	(1.09–6.40)	0.03
All poison excluding paracetamol & hydrocarbon	479/1243 (39)	534/1281 (42)	**2.36**	(1.09–5.15)	0.03	**2.67**	(1.17–6.10)	0.02
Other & unknown poison	73/339 (22)	118/413 (29)	**2.61**	(1.22–5.59)	0.01	**3.43**	(1.60–7.31)	0.001
Paracetamol	35/122 (29)	57/156 (37)	**2.05**	(0.83–5.05)	0.12	**2.41**	(1.06–5.51)	0.04
Hydrocarbon	8/162 (5)	18/170 (11)	**2.21**	(0.65–7.57)	0.21	**1.85**	(0.61–5.65)	0.28
**Forced emesis**	977/1625 (60)	900/1699 (53)	**0.81**	(0.58–1.14)	0.23	**0.99**	(0.80–1.24)	0.95
**Pralidoxime (organophosphate only n = 439)**	14/224 (6)	13/215 (6)	**0.94**	(0.31–2.89)	0.92	**0.98**	(0.29–3.30)	0.98

# Adjusted for covariates - poison type (except for sub-category of poison types), hospital category.

Methionine was not available during the study period and so could not be assessed as an outcome. The use of pralidoxime for organophosphate poisoning remained very low in both intervention and control hospitals (Table 4). The average intra cluster correlation within matched pairs was 0.04 (range 0 to 0.16) for the main outcomes assessed.

### Secondary Outcomes

There were 544 (34%) patients discharged from control hospitals, while 101 (6%) left against medical advice, 974 (60%) were transferred to referral hospital and 6 (0.4%) died before transfer. In intervention hospitals, 473 (28%) patients were discharged while 106 (6%) left against medical advice, and 1,112 (65.5%) were transferred for secondary care and 8 died (0.5%). The proportion of transfers from intervention hospitals was higher than in control hospitals, but this difference was not statistically significant (OR 1.10, 95% CI 0.66–1.83) (Table 5). The follow-up for transferred patients was only implemented in Anuradhapura district (34 hospitals) due to the lack of records from the referral hospital in Polonnaruwa (12 hospitals). In Anuradhapura district, 90 (5.6%) of the transferred patients died in the referral hospitals, and 49 of them were from intervention and 41 from control hospitals, OR 0.97 (95% CI 0.49–1.90) (Table 5).

**Table 5:** Hospital outcome with adjusted odds ratios to assess the effect of the intervention over the 12 months follow-up period in intervention and control hospitals in North Central Province of Sri Lanka.

	Control	Intervention	Adjusted for matched pairs & clustering, Not adjusted for covariates			Adjusted for matched pairs & clustering, & Adjusted for covariates #		
	n (%)	n (%)	OR	95% CI	P value	OR	95% CI	P value
**Peripheral Hospital Outcome**								
Deaths	6 (0.4)	8 (0.5)		–	–		–	–
Transfers	974 (60)	1112 (65)	**1.09**	(0.68–1.76)	0.72	**1.10**	(0.66–1.83)	0.72
Discharged or left against medical advice	645 (40)	579 (34)		ref			ref	
**Secondary Hospital Outcome (after transfer)**								
Deaths	41 (6)	49 (6)	**0.90**	(0.48–1.70)	0.75	**0.97**	(0.49–1.90)	0.92
Discharged or left against medical advice	668 (94)	763 (94)		ref			ref	

# Adjusted for covariates - poison type, hospital category.

The results of above the outcomes at 6 months following intervention were similar results to 12 months analysis (Table S2).

### Validation of the Outcome Measurement with Data Linkage

The agreement between the peripheral hospital record and the transferred patients’ recall of activated charcoal use was very good (367/427 patients, Kappa: 0.75) confirming that there was a real increase in activated charcoal use after the intervention. A post-hoc analysis also showed increased activated charcoal use as recorded by the referral hospital (aOR: 4.1, 95% CI: 1.63 to 10.46).

### Antidote Stocking Practices

The availability of pralidoxime was low and methionine was not available in both intervention and control hospitals due to a national shortages in supplies.

The availability of any stock of activated charcoal in the intervention hospitals was higher (20/23) compared to the control hospitals (15/23) three months after the start of the intervention. This improved to 21/23 intervention and 18/23 control hospitals 6 months later. The data on exact quantities of charcoal stocked was only available from Anuradhapura district (34 hospitals). In this sub group, more intervention hospitals had at least 6 packs of charcoal (the poisoning admissions/month) three months following the intervention [10/17 vs 3/17 (p = 0.002)].

AChE levels were not available for most patients and therefore proposed comparison in organophosphate poisonings was not performed. It had been anticipated that primary hospital use of methionine would have reduced the need for patients to be transferred to secondary hospitals following paracetamol poisoning. Inter-hospital transfers and antidote costs for paracetamol poisoning had been identified as the major driver of costs [28], [29]. As methionine was not available and there was no change in transfer rates the planned cost analysis was not performed.

### Participation in Workshops and Staff Transfers

On average, 70% of staff from intervention hospitals participated in the workshops. The majority of the 30% who did not participate were non-clinical staff. There was some staff turnover during the study period, with a total of 12 doctors transferred out of the study hospitals, −2 from intervention hospitals and 10 from controls. Only one doctor was transferred from an intervention to a control hospital and two doctors transferred from control hospitals to intervention hospitals. Six doctors (including 4 graduates) were transferred into intervention hospitals, and 12 doctors (8 new graduates) were transferred into control hospitals. There were 31 nurse transfers during the study period, but only 4 transferred from an intervention to a control hospital.

## Discussion

This randomized controlled trial showed that a brief academic detailing intervention delivered in peripheral hospitals was effective in increasing use of activated charcoal. Administration of activated charcoal for poisoned patients was one of the key messages of the intervention and under the direct control of the clinician. This improvement indicated that academic detailing interventions are effective in translating guidelines into practice. In contrast, there were no significant changes for the gastric emptying treatments (such as forced emesis) that were usually initiated soon after admission by non medical staff. Although an overall increase in charcoal use was intended, the wall-chart, which was provided as a part of the intervention (Figure 1), provided differing guidance based on poison types. For example, charcoal use is contra-indicated for patients who have ingested hydrocarbons, and not recommended in paracetamol poisoning if methionine is to be given. Thus the increase in use across several different poison types (Table 4) in intervention hospitals reinforces the conclusion that the increase in use was appropriate. The similar odds ratios for charcoal use at 6 and 12 months suggest a sustained change in treatment behavior following the intervention.

These results emphasize the importance of broadening the focus of education programs by involving all staff that are involved in patient care. In this study, the majority (70%) of staff members who were involved with poisoning treatments in the intervention hospitals received the education intervention. The participation of doctors was higher than nursing and non-professional staff. The higher level of participation by doctors may have contributed to the greater changes in their treatment practices, as it is important that participants feel as if they “own” the change [4]. More specific or prolonged approaches through integrated educational programs would be useful in reaching all staff members.

Poisoning in Sri Lanka is predominately in the context of deliberate self-harm and is a highly emotive clinical condition involving many staff members, the patient, and their family. These patients require urgent assessment and treatment often in a setting of incomplete information about the exposure history and the precipitating incidents. Treatment is often carried out in the presence of family and community members who may influence treatment decisions creating a complex clinical treatment scenario and a challenging task for staff. It is likely that any educational strategy that can influence treatment behavior in difficult clinical situations such as poisoning could be effective in translating other clinical guidelines for less acute and less charged clinical situations in similar settings.

### Strengths and Limitations

This study included all the peripheral hospitals in the province and allowed observations of treatment behavior changes of a complete health administrative area. The number of patients who were transferred out of this province was low and could therefore not affect the conclusions of the study. Furthermore, the geographic separation of the intervention and control hospitals was sufficient to minimize the risk of contamination. Hospitals generally transferred patients directly to central secondary care hospitals rather than closer but larger peripheral hospitals. These admission and referral patterns allowed examination to focus on primary admissions and to validate a large subset of patients’ peripheral hospital record by direct interviews with transferred patients.

The number of deaths was too small (around 3.1%) to provide sufficient statistical power to see an effect of the intervention. A future study would have to be about three times larger to detect a one third reduction in deaths. Improved case-fatality to this extent would also likely require increased use of specific antidotes. The intervention included messages about maintaining appropriate antidote stocks. All study hospitals had the same level of access to ordering antidotes. While most intervention and control hospitals stocked charcoal, intervention hospitals were more likely to hold appropriate charcoal stocks suggesting a change in the ordering pattern. As this was an intention to treat analysis there was no adjustment for charcoal availability. Hospitals had to both order and use charcoal to respond to the intervention. However, other antidotes, such as pralidoxime and methionine were largely unavailable in both intervention and control hospitals due to local shortages. Hence, this study was not able to assess whether the intervention could improve antidote usage for organophosphate and paracetamol poisonings. The educational intervention advised that activated charcoal should not be administered to patients with paracetamol poisoning. This was because activated charcoal would not reduce the need to administer the specific antidote methionine and could interfere with methionine absorption. This advice was supported by comprehensive written guidelines and the wall chart. The results showed increased use of charcoal for paracetamol poisonings in the intervention group, although this was less than the increase for other poisonings. In the absence of methionine, charcoal administration is clinically appropriate. As methionine was unavailable and the supply of activated charcoal improved (Table 4) it is possible that staff made a decision to use charcoal based upon the educational intervention’s written guidelines or presentation. Situations like this should be further assessed in future research using post-intervention surveys to understand participant’s changes in practice when the first-line recommended treatments are not available.

There was incomplete participation of staff in the intervention hospitals in the academic detailing workshops, which may have reduced the effect of the intervention. On average only 70% of staff members participated due to shift work and holidays. Train the trainer or follow-up programs to deliver the intervention to the group who did not participate may have improved the effectiveness but would increase the resources required to deliver the intervention.

During the 12 month data collection period, a small number of staff members from both intervention and control hospitals transferred in and out of hospitals. These transfers would have potentially reduced differences between the treatment arms. The number of admissions to base and district hospitals in the control group was higher than the intervention group (Table 2). This difference might have generated bias and masked the true effect of the intervention. The random effect model which adjusts odds ratios for hospital category should have minimized this effect.

In Polonnaruwa district we were unable to compare the peripheral hospital records with the data collected from the secondary hospital, thus limiting our capacity to validate the peripheral hospital records in that district. This also reduced the data available to assess secondary outcomes.

### Applicability of Out-reach Academic Detailing Approach

The most commonly used methods to promote treatment guidelines in Sri Lanka are centralized programs and distribution of printed books [6], [7], [10]. This study indicates that providing a brief educational intervention in peripheral hospitals combined with printed guidelines was more effective than distributing printed guidelines alone. The intervention required a substantial time commitment by the providers but decreased the logistic and practical barriers for the recipients leading to higher and broader staff participation rates.

Out-reach academic detailing originated from the strategies used by pharmaceutical companies in promoting their products. This method is recognized as an effective way of influencing clinical decision making and treatment behavior [22]. The study intervention has similar characteristics to out-reach academic detailing in previous studies [21], [30], [31], [32]. The techniques used in academic detailing include assessing current practices, focusing on specific categories or opinion leaders, defining behavioral objectives, establishing credibility through organization identity or source of information, stimulation through interactions, using graphical educational materials, highlighting essential messages and providing positive reinforcement [22]. In the intervention many of the above techniques were used including: a focus on specific categories, namely the peripheral hospital staff; defining clear behavioral change objectives, such as improving poisoned patient management; establishing credibility by using national poisoning treatment guidelines; and with a partnership of provincial health authorities, using graphical educational materials, such as wall charts and highlighting and repeating messages using promotional items (Figure 1). Hence this cluster RCT provides evidence to support the application of an out-reach academic detailing approach in promoting clinical guidelines in rural hospital settings in Sri Lanka and other similar regions.

### Further Research and Implications

There was no difference in the number of transfers from either intervention or control hospitals to the secondary care hospital. Previous studies have highlighted that patient transfer, especially poisoned patients, is not only dependant on the patient clinical condition but is also a result of long established hospital culture and the influence of the family and relatives of the patient [11]. An intervention which only targets hospital staff may not be effective in changing such practices. Future research incorporating community awareness programs as well as hospital education interventions are needed to assess if changes of community awareness will help improve treatment outcomes in this setting.

Our study showed the intervention was effective in changing treatments authorized by a doctor, but not treatments usually initiated by non-professional staff. Understanding the factors that influence these staff’s treatment practice is needed to help design future interventions to influence their behavior.

Examination of other methods of training delivery to peripheral hospitals such as training local trainers could help address the sub-optimal participation rate and also might empower staff to have appropriate local adaptation of guidelines.

### Conclusion

This cluster randomized controlled trial showed that a brief academic detailing educational intervention was effective in improving poisoned patient care in a peripheral hospital setting. But this effect was only for the treatments with direct clinician involvement, such as administering activated charcoal. The intervention was not successful for treatments usually administered by non-professional staff such as forced emesis for poisoning. This latter group was less engaged in the education and also may be more subject to external community pressures. Specific interventions targeting the community and non-medical staff and practices may be useful to change practice in similar settings.

## Supporting Information

Table S1 **Primary outcomes with adjusted odds ratios over the 6 and 12 months follow-up period.**
(DOCX)

Table S2 **Secondary outcomes with adjusted odds ratios over the 6 and 12 months follow-up period.**
(DOCX)

Checklist S1 **CONSORT Checklist.**
(DOCX)

Protocol S1 **Trial Protocol.**
(PDF)
