# Supplementary material for: Effect of a Brief Outreach Educational Intervention on the Translation of Acute Poisoning Treatment Guidelines to Practice in Rural Sri Lankan Hospitals: A Cluster Randomized Controlled Trial
Source: PLoS One. 2013 Aug 19;8(8):e71787. doi: 10.1371/journal.pone.0071787 (PMC3747188; doi:10.1371/journal.pone.0071787)
Supplement: Table S1 — Primary outcomes with adjusted odds ratios over the 6 and 12 months follow-up period. (DOCX) [file pone.0071787.s001.docx]

**Table S1:** Primary outcomes with adjusted odds ratios to assess the effect of intervention over the 6 and 12 months follow-up period in intervention and control hospitals in North Central Province of Sri Lanka

|  | **aOR – Adjusted for clustering, pairing** | **95% CI** | **P Value** | **aOR – Adjusted for clustering, pairing & covariates^#^** | **95% CI** | **P Value** |
| --- | --- | --- | --- | --- | --- | --- |
| **Activated Charcoal - Overall** |  |  |  |  |  |  |
| For first 6 Months | 2.30 | 1.04 - 5.07 | 0.04 | 2.83 | 1.18 - 6.78 | 0.02 |
| For 12 months | 2.29 | 1.04 - 5.07 | 0.04 | 2.95 | 1.28 - 6.80 | 0.01 |
| **Activated Charcoal - Pesticides** |  |  |  |  |  |  |
| For first 6 Months | 2.22 | 1.00 5.00 | 0.05 | 2.54 | 1.08 5.95 | 0.03 |
| For 12 months | 2.37 | 0.96 - 5.85 | 0.06 | 2.64 | 1.09 - 6.40 | 0.03 |
| **Activated Charcoal - All poison excluding: paracetamol, hydrocarbon** |  |  |  |  |  |  |
| For first 6 Months | 2.35 | 1.07 - 5.14 | 0.03 | 2.77 | 1.23 - 6.24 | 0.01 |
| For 12 months | 2.36 | 1.09 - 5.15 | 0.03 | 2.67 | 1.17 - 6.10 | 0.02 |
| **Activated Charcoal - Other & unknown poison** |  |  |  |  |  |  |
| For first 6 Months | 2.22 | 1.03 4.76 | 0.41 | 2.61 | 1.15 - 5.89 | 0.02 |
| For 12 months | 2.61 | 1.22 - 5.59 | 0.01 | 3.43 | 1.60 - 7.31 | 0.001 |
| **Activated charcoal - Paracetamol** |  |  |  |  |  |  |
| For first 6 Months | 2.34 | 1.09 - 5.03 | 0.03 | 2.66 | 1.15 - 6.16 | 0.02 |
| For 12 months | 2.05 | 0.83 - 5.05 | 0.12 | 2.41 | 1.06 - 5.51 | 0.04 |
| **Forced emesis** |  |  |  |  |  |  |
| For first 6 Months | 0.66 | 0.41 - 1.07 | 0.09 | 0.78 | 0.52 1.18 | 0.24 |
| For 12 months | 0.81 | 0.58 - 1.14 | 0.23 | 0.99 | 0.80 - 1.24 | 0.95 |
| **Pralidoxime** |  |  |  |  |  |  |
| For first 6 Months | 0.93 | 0.28 - 3.12 | 0.91 | 0.98 | 0.26 - 3.79 | 0.98 |
| For 12 months | 0.94 | 0.31 - 2.89 | 0.92 | 0.98 | 0.29 3.30 | 0.98 |

# Adjusted for covariates - poison type (except for sub-category of poison types), hospital category
