# Supplementary material for: Effect of a Brief Outreach Educational Intervention on the Translation of Acute Poisoning Treatment Guidelines to Practice in Rural Sri Lankan Hospitals: A Cluster Randomized Controlled Trial
Source: PLoS One. 2013 Aug 19;8(8):e71787. doi: 10.1371/journal.pone.0071787 (PMC3747188; doi:10.1371/journal.pone.0071787)
Supplement: Table S2 — Secondary outcomes with adjusted odds ratios over the 6 and 12 months follow-up period. (DOCX) [file pone.0071787.s002.docx]

**Table S2:** Secondary outcomes with adjusted odds ratios to assess the effect of intervention over the 6 and 12 months follow-up period in intervention and control hospitals in North Central Province of Sri Lanka

|  | **aOR – Adjusted for clustering, pairing** | **95% CI** | **P Value** | **aOR – Adjusted for clustering, pairing & covariates^#^** | **95% CI** | **P Value** |
| --- | --- | --- | --- | --- | --- | --- |
| **Peripheral hospital outcome - transfer** |  |  |  |  |  |  |
| For first 6 Months | 0.92 | 0.56 - 1.53 | 0.75 | 0.94 | 0.55 - 1.60 | 0.82 |
| For 12 months | 1.09 | 0.68 - 1.76 | 0.72 | 1.10 | 0.66 - 1.83 | 0.72 |
| **Secondary hospital outcome - Deaths** |  |  |  |  |  |  |
| For first 6 Months | 0.52 | 0.23 - 1.17 | 0.11 | 0.46 | 0.19 - 1.11 | 0.08 |
| For 12 months | 0.90 | 0.48 - 1.70 | 0.75 | 0.97 | 0.49 - 1.90 | 0.92 |

# Adjusted for covariates - poison type (except for sub-category of poison types), hospital category
