# Supplementary material for: Effect of a Brief Outreach Educational Intervention on the Translation of Acute Poisoning Treatment Guidelines to Practice in Rural Sri Lankan Hospitals: A Cluster Randomized Controlled Trial
Source: PLoS One. 2013 Aug 19;8(8):e71787. doi: 10.1371/journal.pone.0071787 (PMC3747188; doi:10.1371/journal.pone.0071787)
Supplement: Checklist S1 — CONSORT Checklist. (DOCX) [file pone.0071787.s003.docx]

**Effect of a brief outreach educational intervention on the translation of acute poisoning treatment guidelines to practice in rural Sri Lankan hospitals: A cluster randomized controlled trial.**

Lalith Senarathna, Nick A Buckley, Michael J Dibley, Patrick J Kelly, Shaluka F Jayamanna, Andrew H Dawson

CONSORT 2010 checklist of information to include when reporting a randomised trial*

| Section/Topic | Item No | Checklist item | | | | Reported on section | |
| --- | --- | --- | --- | --- | --- | --- | --- |
| Title and abstract | | | | | | | |
|  | 1a | Identification as a randomised trial in the title | | | Title | | |
|  | 1b | Structured summary of trial design, methods, results, and conclusions (for specific guidance see CONSORT for abstracts [1, 2]) | | | Abstract | | |
| Introduction | | | | | | | |
| Background and objectives | 2a | Scientific background and explanation of rationale | | | Introduction | | |
|  | 2b | Specific objectives or hypotheses | | | Introduction | | |
| Methods | | | | | | | |
| Trial design | 3a | Description of trial design (such as parallel, factorial) including allocation ratio | Introduction – Study Design & Setting | | | | |
|  | 3b | Important changes to methods after trial commencement (such as eligibility criteria), with reasons | N/A | | | | |
| Participants | 4a | Eligibility criteria for participants | Methods - Study Setting & Design | | | | |
|  | 4b | Settings and locations where the data were collected | Methods - Study Setting & Design | | | | |
| Interventions | 5 | The interventions for each group with sufficient details to allow replication, including how and when they were actually administered | Methods – Intervention | | | | |
| Outcomes | 6a | Completely defined pre-specified primary and secondary outcome measures, including how and when they were assessed | Methods – Data Collection | | | | |
|  | 6b | Any changes to trial outcomes after the trial commenced, with reasons | Results – Primary Outcomes | | | | |
| Sample size | 7a | How sample size was determined | Methods – Sample Size | | | | |
|  | 7b | When applicable, explanation of any interim analyses and stopping guidelines | N/A | | | | |
| Randomisation: |  |  |  | | | | |
| Sequence generation | 8a | Method used to generate the random allocation sequence | Methods – Randomization | | | | |
|  | 8b | Type of randomisation; details of any restriction (such as blocking and block size) | Methods – Randomization | | | | |
| Allocation concealment mechanism | 9 | Mechanism used to implement the random allocation sequence (such as sequentially numbered containers), describing any steps taken to conceal the sequence until interventions were assigned | N/A | | | | |
| Implementation | 10 | Who generated the random allocation sequence, who enrolled participants, and who assigned participants to interventions | Methods – Randomization | | | | |
| Blinding | 11a | If done, who was blinded after assignment to interventions (for example, participants, care providers, those assessing outcomes) and how | N/A | | | | |
|  | 11b | If relevant, description of the similarity of interventions | N/A | | | | |
| Statistical methods | 12a | Statistical methods used to compare groups for primary and secondary outcomes | Methods - Statistical analysis | | | | |
|  | 12b | Methods for additional analyses, such as subgroup analyses and adjusted analyses | Methods - Statistical analysis | | | | |
| Results | | | | | | | |
| Participant flow  (a diagram is strongly recommended) | 13a | For each group, the numbers of participants who were randomly assigned, received intended treatment, and were analysed for the primary outcome | Results & Figure 2 | | | | |
|  | 13b | For each group, losses and exclusions after randomisation, together with reasons | N/A | | | | |
| Recruitment | 14a | Dates defining the periods of recruitment and follow-up | Results | | | | |
|  | 14b | Why the trial ended or was stopped | Results | | | | |
| Baseline data | 15 | A table showing baseline demographic and clinical characteristics for each group | Table 2 | | | | |
| Numbers analysed | 16 | For each group, number of participants (denominator) included in each analysis and whether the analysis was by original assigned groups | Results | | | | |
| Outcomes and estimation | 17a | For each primary and secondary outcome, results for each group, and the estimated effect size and its precision (such as 95% confidence interval) | Results – Primary Outcomes & Secondary Outcomes | | | | |
|  | 17b | For binary outcomes, presentation of both absolute and relative effect sizes is recommended | Results – Primary Outcomes & Secondary Outcomes | | | | |
| Ancillary analyses | 18 | Results of any other analyses performed, including subgroup analyses and adjusted analyses, distinguishing pre-specified from exploratory | Results - Validation of the outcome measurement with data linkage | | | | |
| Harms | 19 | All important harms or unintended effects in each group (for specific guidance see CONSORT for harms^28^) | N/A | | | | |
| Discussion | | | | | | | |
| Limitations | 20 | Trial limitations, addressing sources of potential bias, imprecision, and, if relevant, multiplicity of analyses | | Discussion- Strengths and limitations | | | |
| Generalisability | 21 | Generalisability (external validity, applicability) of the trial findings | | Discussion - Further research and implications | | | |
| Interpretation | 22 | Interpretation consistent with results, balancing benefits and harms, and considering other relevant evidence | | Discussion & Conclusions | | | |
| Other information | | | |  | | | |
| Registration | 23 | Registration number and name of trial registry  *ISRCTN73983810*  *Current Controlled Trials* | |  | | | |
| Protocol | 24 | Where the full trial protocol can be accessed, if available  *Full Protocol is attached as a supporting document ( Protocol S1)* | |  | | | |
| Funding | 25 | **Sources of funding and other support (such as supply of drugs), role of funders**  *The South Asian Clinical Toxicology Research Collaboration funded this study (Wellcome Trust/National Health and Medical Research Council International Collaborative Research Grant GR071669MA and NHMRC GNT0630650). Lalith Senarathna was supported by the Australian Endeavour Postgraduate Award. None of the funding sources had any role in study design, in data collection, analysis or interpretation, or in the preparation or publication of this paper.* | | | | |  |

*We strongly recommend reading this statement in conjunction with the CONSORT 2010 Explanation and Elaboration [3] for important clarifications on all the items. If relevant, we also recommend reading CONSORT extensions for cluster randomised trials, [4] non-inferiority and equivalence trials [5], non-pharmacological treatments [6], herbal interventions [7], and pragmatic trials [8]. Additional extensions are forthcoming: for those and for up to date references relevant to this checklist, see [www.consort-statement.org](http://www.consort-statement.org).

References

1. Hopewell S, Clarke M, Moher D, Wager E, Middleton P, Altman DG, et al. CONSORT for reporting randomised trials in journal and conference abstracts. *Lancet* 2008;371:281-3.
2. Hopewell S, Clarke M, Moher D, Wager E, Middleton P, Altman DG, et al. CONSORT for reporting randomized controlled trials in journal and conference abstracts: explanation and elaboration. *PLoS Med* 2008;5:e20.
3. Moher D, Hopewell S, Schulz KF, Montori V, Gøtzsche PC, Devereaux PJ, et al. CONSORT 2010 Explanation and Elaboration: updated guidelines for reporting parallel group randomised trials. *BMJ* 2010;340:c869.
4. Campbell MK, Elbourne DR, Altman DG. CONSORT statement: extension to cluster randomised trials. *BMJ* 2004;328:702-8.
5. Piaggio G, Elbourne DR, Altman DG, Pocock SJ, Evans SJ. Reporting of noninferiority and equivalence randomized trials: an extension of the CONSORT statement. *JAMA* 2006;295:1152-60.
6. Boutron I, Moher D, Altman DG, Schulz KF, Ravaud P. Extending the CONSORT statement to randomized trials of nonpharmacologic treatment: explanation and elaboration. *Ann Intern Med* 2008;148:295-309.
7. Gagnier JJ, Boon H, Rochon P, Moher D, Barnes J, Bombardier C. Reporting randomized, controlled trials of herbal interventions: an elaborated CONSORT statement. *Ann Intern Med* 2006;144:364-7.
8. Zwarenstein M, Treweek S, Gagnier JJ, Altman DG, Tunis S, Haynes B, et al. Improving the reporting of pragmatic trials: an extension of the CONSORT statement. *BMJ* 2008;337:a2390
