# Supplementary material for: Effect of a Brief Outreach Educational Intervention on the Translation of Acute Poisoning Treatment Guidelines to Practice in Rural Sri Lankan Hospitals: A Cluster Randomized Controlled Trial
Source: PLoS One. 2013 Aug 19;8(8):e71787. doi: 10.1371/journal.pone.0071787 (PMC3747188; doi:10.1371/journal.pone.0071787)
Supplement: Protocol S1 — Trial Protocol. (PDF) [file pone.0071787.s004.pdf]

Title:

A hospital based cluster randomised controlled trial to evaluate treatment behaviour following educational interventions and essential antidote stocking in the treatment of acute poisoning patients.

Short Title:

Evaluation of Primary Hospital Education Intervention

Lalith Senarathna, Shaluka Jayamanna, Nick A Buckley, Indika Gawarammana, Michael J Dibley and Andrew Dawson

South Asian Clinical Toxicology Research Collaboration (SACTRC)
Faculty of Medicine
University of Peradeniya
Peradeniya
Sri Lanka

Overview:

Acute poisoning is a major health issue around the world. But the mortality and morbidity figures are higher in developing countries comparing to the industrialized countries. The main reason was explained as the type of poisons ingested. Many developing countries, ingesting pesticides and plant residuals have become the common methods resulting mortality a twenty times higher than industrialized countries where ingesting pharmaceuticals is common. In rural agricultural districts of Sri Lanka acute poisoning has become a significant cause of hospital admissions. This situation is more prominent in the North Central Province of Sri Lankan where the second highest cause of hospitals deaths is poisoning in recent years [1-5].

Treatments for acute poisoning patients were initially provided in smaller peripheral hospitals and patients are then transferred to larger referral hospitals. North Central Province of Sri Lanka has an overall case fatality ratio of 9% and it may be influenced by the availability of treatment facilities, antidotes, staff and utilization of treatment protocols (based on knowledge and attitude). Previous studies [6-8] have examined the association of the availability of proper treatment protocols, availability of appropriate antidotes and other medication and equipments and staff attitude with patient outcome in rural primary care hospitals.

This study is planned as a cluster randomized control trial to assess the effectiveness of an education intervention on treating poisoned patients. The study results would be important in understanding the changes of the treatment behavior of peripheral hospital staff following an educational intervention.

Aims:

The main aim of the study is to use cluster randomised controlled trial design to assess the effectiveness of an educational intervention on poisoning treatment with essential antidote list to change the treatment behaviours of primary hospital (peripheral hospital) staff for treating poisoned patients, to establish effectiveness of low cost education program in low resource set up to change staff treatment behaviour and to examine the duration of effects after the intervention.

Primary Hypothesis:

In a hospital based cluster randomised controlled trial of staff from peripheral hospitals in the North Central Province of Sri Lanka, an educational intervention based on poisoning patient management and antidote utilisation will increase the use of single dose of activated charcoal for poisoned patients who presented within 2 hours of ingestion from 35% to 50% compared with the control group of hospitals.

Secondary Hypothesis:

- The percentage of poisoning patients who receive forced emesis as a decontamination method will reduce in the interventional peripheral hospitals compared with the control peripheral hospitals.

- The percentage of poisoned patients who receive gastric lavage after the recommended time of the guidelines/intervention will reduce in the intervention peripheral hospitals compared with the control peripheral hospitals.

- The percentage of organophosphate pesticide ingested patients who receive single dose of pralidoxime will increase in the intervention peripheral hospitals compared with the control peripheral hospitals.

- The percentage of poisoned patients who are transferred to secondary care hospitals from interventional peripheral hospitals will be reduced compared with the control peripheral hospitals.

- The percentage of poisoned patients who die either in the primary care or secondary care or tertiary care after initially admitted to interventional peripheral hospital will decrease compared with the control peripheral hospitals.

- The average score from the data recording quality assessment score (Appendix 1) of the interventional peripheral hospitals will be higher than the control peripheral hospitals.

- The monthly average stock and the number of requests for new stocks of essential antidotes in terms of activated charcoal, pralidoxime and atropine will increase in the interventional peripheral hospitals compared with the control interventional hospitals.

Background

Deliberate self poisoning is a major public health problem around the world. Comparing to the mortality from poisoning in industrialized countries, the rates are approximately twenty times higher in developing countries [1]. The higher mortality figures could be highly attributed to the availability of highly toxic substances such as pesticides I these [9]. A survey study conducted among poisoned patients who admitted to a secondary hospital in Sri Lanka, many patients were young with median age 25 years and mostly males deliberately ingested poisonous substances. The main type of poison used was pesticides and majority of male patients had ingested pesticides while higher percentage of females has ingested yellow oleander seeds which contain cardiac glycosides. Although the overall mortality was 9%, there were more deaths among male patients comparing to female patients (12.4 Vs 4.5). And also more female deaths occurred in age group of below 25 years and male deaths were more evenly distributed with 22% deaths in the group below 25 years [5].

Another survey conducted among hospitalized poisoned patients in North Central Province of Sri Lanka revealed that majority of patients (85%) selected the poison based on the easy availability. And also, there were little premeditation, more than 50% ingested poison less than 30 minutes after deciding to self harm [10].

A recent case study and literature review titled " Pesticide poisoning: a major health problem in Sri Lanka" [8] attempted to analyze the reasons for the high number of poisoning incidences and deaths. Apart from the reasons for attempts, the importance of the availability of medical facilities to treat poisoning patients were highlighted in the paper. It also highlighted that there were no antidotes available in rural hospitals to treat poisoning patients and this might be the reason for the high death rates.

A prospective study in rural Sri Lanka which aimed to determine the patterns of patient transfer from rural hospitals to secondary or tertiary care hospitals [11], found that nearly 50% of poisoning patients admitted to rural hospitals were being treated and discharged from the same hospital. The distance from the secondary care centre may have acted as a confounder in this study, nonetheless these study findings revealed that rural hospitals play a major role in the management of poisoned patients from rural areas in Sri Lanka, a situation also likely in other developing countries.

The relationship between availability of resources in terms of antidotes, equipments and staff in peripheral hospitals in north central province of Sri Lanka and patient outcome was assessed in a cross-sectional study in 2006 [6]. The study revealed that many hospitals in these rural areas do not have adequate antidotes, equipments or trained staff. It had resulted transferring large number of patients from peripheral hospitals to secondary hospitals without receiving proper initial treatment. And also the unavailability and lack of adherence to a proper treatment protocol was also responsible for higher morbidity, there is a significant mismatch between what hospital staff believe as what they should do and what they actually practice. It emphases the importance of the availability of a standard treatment protocol together with adequate supply of antidotes to the peripheral hospitals.

Another recent study had explored the personal and professional challenges faced by rural hospitals doctors when treat poisoned patients [7]. Apart from the lack of resources, more doctors had revealed that professional isolation and pressure from community or relatives of the patients are main challenges during their practice. The professional isolation which caused by the lack of continuous education and limited access to the updated knowledge could resulted less confident when work in a low resource hospital. The study concluded that taking measures to address the issue of professional isolation would be an economical and sustainable way to improve the poisoning patient care in peripheral hospital level.

Providing education to the staff of low resource hospital is met with various challenges. The unavailability of adequate staff is a main barrier for conducting conventional centralized workshops. Many hospital staff has limited opportunity to attend as they would have to close the hospital if they come for a workshop. This

knowledge triggers that de-centralized educational programs as out-reach visits may benefit the low resource peripheral hospital set as concluded in previous studies done in other countries [12].

Few studies were done in western hospital set up to promote clinical guidelines of other specialties through non-conventional continuous education methods had found that there is a possibility to change the clinical behavior of doctors, but emphases that more effort should be put on the longer interventions which provide extended support. And also continuous feedback and professional support along the way were also seen as important [13-15].

A pilot study had conducted in peripheral hospitals in Sri Lanka by randomizing hospital in to intervention and control hospitals to receive a single lecture with supporting materials like wall charts and no special lectures respectively. This study had demonstrated the extreme diversity of basic management practices and the need for analyzing in any definitive study that accounts for clustering and had shown that the of both baseline and effects of the change [16].

We, planned to do a cluster randomized controlled trial to deliver an education intervention based on the national guideline of poisoning management together with continuous supply of essential antidotes, promotional items to improve the adherence and with a continuous monitoring system. The main outcome measure is the changes of the treatment behavior which will be assessed related to specific treatments and procedures.

Significance

Acute poisoning with pesticides and other hazard plant residues has reached an epidemic level in Sri Lanka. This situation is similar to that in other developing countries where small scale agricultural systems prevail. The Sri Lankan national guideline of treating poisoning patients emphasizes that poisoned patients should be treated according to the recommendations after they present to a health care facility. Therefore providing education and training for the staff is important in reducing mortality and morbidity from acute poisonings. This study will use the cluster randomized control trial design to introduce educational intervention with continuing supply of essential three common antidotes (activated charcoal, pralidoxime, methionine) to assess the changes of the treatment behaviours of primary hospital staff for treating poisoned patients in terms of gastrointestinal decontamination, use of

antidotes and transferring for secondary care. The results will be the effectiveness of the intervention on initiating behavioural changes and the sustainability of the change. And also the effect of this outreach intervention towards the attitude of the hospital staff will be explored. All together the results will not be only limited to improving poisoned patient care in peripheral hospital level but also open an opportunity to create a set of generic procedures to promote guidelines in low resource hospital setup.

The Methodology of the study:

Study design:

This study is planned as an experimental study with cluster randomized control design. Each of the peripheral hospital in the study area is considered as a cluster.

The randomization will be done to produce two arms; intervention arm to receive an education intervention (see the intervention plan) and a control arm.

The study area/Population:

The cluster RCT will be conducted in the primary care hospitals (based on the Sri Lankan ministry of health categorization) in North Central Province of Sri Lanka. Base hospitals, district hospitals, peripheral units, rural hospitals (Figure 1) will be included in this category and central dispensaries will not be considered as there are no in-patient care facilities for poisoned patients.

The staff of these hospitals consists of doctors, nurses, attendants (similar to nursing assistants without an academic training), pharmacists/dispensers, labourers, ambulance drivers. All these staff members will be included in the study and asked to participate for the short workshops – the part of the intervention.

Figure 1: Types of hospitals to be considered for the study.

Base hospitals
District hospitals } **Included in the study**
Peripheral units
Rural hospitals
Central dispensaries

During the study all above mentioned hospital types will be considered as peripheral hospitals or primary care hospitals.

Recruitment criteria for hospitals:

There are 46 hospitals in the province (study area) with in-ward care facilities. All these hospitals which can provide inward care for poisoned patients were eligible for the study. It makes the total number of participating hospitals 46.

The bed strength of these hospitals vary from 11 to 160 and the annual number of admission (due to all causes) to those hospital vary 700 to 13 000. This ensures that the selected group of hospitals represents different levels of the peripheral hospitals.

Sampling design:

The all 46 hospitals with in-patient facilities were considered as eligible for the study and included. 23 hospitals will be allocated to treatment & control.

Allocation to intervention/control groups:

The number of beds available in wards (bed strength) and annual number of admissions from all causes will be recorded for each hospital. Then each hospital is ranked based on the bed number. When there is more than one hospital with the same bed number, same rank will be given to all. The same ranking system will be applied to the annual admissions (all causes) of eligible hospitals. The average rank of bed strength and annual admissions per each hospital will be calculated and hospitals will be arranged according to the ascending order of average rank. After that the adjoining hospitals from the sorted list will be paired and those pairs will be used for randomization.

Finally one hospital will be selected from the paired hospitals as the intervention hospital. The other hospital will remains as the control hospital.

Study Interventions:

The study intervention contains educational intervention as a short workshop on poisoning patient management, antidote administration and stabilization of poisoning patients which is to be delivered by a senior consultant physician. All these workshops are conducted as academic detailing sessions in respective hospitals (as

outreach). The content and the key messages of the educational intervention were from the standard national poisoning treatment guideline [17].

A continuous supply of antidotes (activated charcoal (all poison types except acid/alkaline, heavy metals and hydrocarbon), pralidoxime (organophosphate poisoning) to the all study hospitals will be assured as a part of the study. These hospitals are encouraged to report any out of stock item immediately as a part of the intervention. The government procedure of supplying antidotes to hospitals will not be altered hence control hospitals will have access to all antidotes throughout the study period.

-Reducing forced emesis and performing gastric lavage when only appropriate
(reduce using gastric lavage as a routine procedure).
-Administering Activated charcoal
-Administering Pralidoxime

Educate the hospitals doctors and pharmacists on appropriate antidote management.

-Storing appropriate quantity of antidotes considering the epidemiology of the poisoning.
-Administering antidotes appropriately.
-Maintaining appropriate antidote stocks and proper ordering and storing procedures.

In addition to the educational intervention, all interventional hospitals receive promotional items such as pens, cardboard folders and notice boards with messages about following guidelines discussed in the educational intervention.

The control hospitals will also receive copies of the national guidelines of poisoning management [17] and supply of antidotes from the usual routine supply.

Delivering study intervention:

The educational intervention about poisoning patient management is planned as an outreach program and will be delivered in the respective hospitals as single, participatory workshop by a senior clinician.

All intervention hospital staff members will be asked to participate for the program. A lecture using PowerPoint slides will be used to introduce the key messages and then participants were allowed to ask questions leading to discussion on the difficulties, challenges and queries.

All intervention hospitals will receive display boards/wall charts about the standard poisoning patient management protocols (Figure 2). Which is to be displayed in the ward and it gives a comprehensive guide on following guidelines.

All study hospitals will receive promotional items such as notice boards, folders and pens to encourage using national guideline recommendations. Distribution of promotional items will create an environment which encourages the peripheral hospital staff to adhere to the national guideline recommendations.

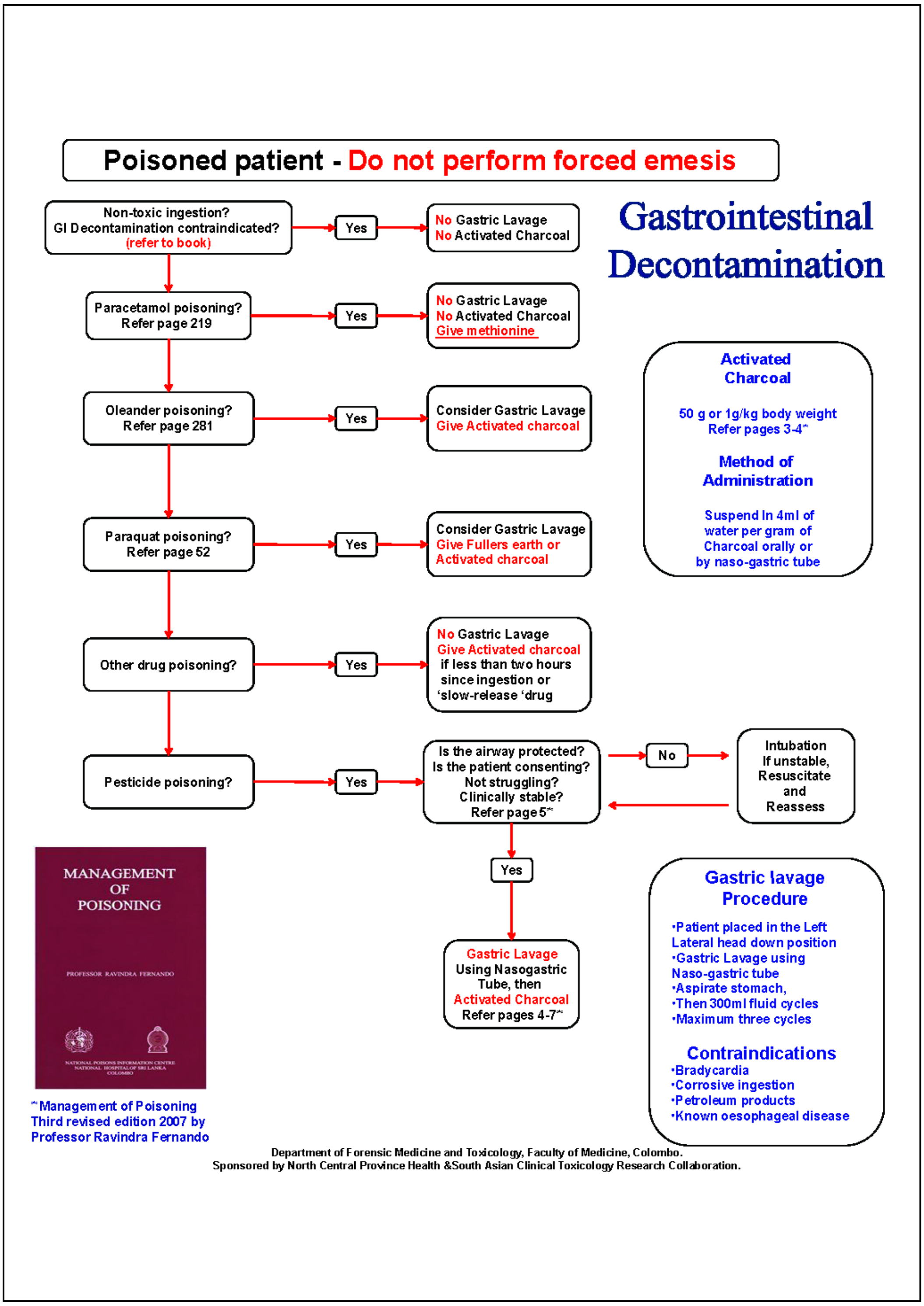

Figure 2: Wall chart to distribute to intervention hospitals

Delivering Antidotes:

For the study hospitals, continuous supply of antidotes; activated charcoal, pralidoxime will be organized. In the beginning of the study, all interventional hospitals will be given one month supply of antidotes. The re-ordering of antidotes

should be done by the hospitals divisional pharmacist. The NCP study office will take care of the continuous supply of antidotes through divisional pharmacist.

Evaluation plan:

All primary hospitals would have repeated visits to conduct a chart review to collect data. Chart review would be performed in the medical records departments that are remote to the clinical wards. Also during this time and during the subsequent trial all patients who had been transferred to the referral hospitals would be seen in the referral hospital and their admission would be linked to their primary hospital admission. The history and signs of primary hospital intervention would be documented at the referral hospital.

These Data collections would be continued by regular review of the medical records to record documented treatments from the primary hospital record. This documented treatment would be validated by obtaining a direct history from transferred patients following arrival in the secondary referral hospitals.

We have ethical approval for and are currently conducting an observational study in the major referral hospitals which would allow us to prospectively record clinical effects of the interventions and to confirm, using history, physical examination and blood analysis, whether or not the documented treatment in the primary hospital was delivered.

Admission and outcome data from the secondary referral hospitals will also be confirmed by auditing the medical record. All extracted individual patient data will be stored in a password-protected database. Stock levels of antidotes and treatments would be collected at the peripheral hospitals and correlated with ordering patterns from the central dispensary

Data Collection:

Data collection will be done by primary researcher and specially trained research assistants. During the study period both quantitative and qualitative data will be collected.

Data will be collected from

1. Poisoned patients – from the patients records for poisoned patients who admitted to study hospitals during study period
2. From the hospital – this will happen in organizational level by reviewing the records, drug stock details, documentation on drug ordering and transferring patients to secondary hospitals.
3. From the secondary transferred hospitals – The patient review charts and records will be used to collect these details.

Outcomes

Primary

1. Use of activated charcoal:
   a. Documentation of primary hospital charts
   b. Validation by history of intervention and examination (for evidence of charcoal ingestion) in a subset transferred to referral hospital

2. Use of forced emesis :
   a. Documentation of primary hospital charts
   b. Validation by history of intervention in the subset transferred to referral hospital

3. Use of pralidoxime:
   a. Documentation of primary hospital charts
   b. Validation by hospital pharmacy records and central pharmacy ordering. Analytical confirmation in the subset transferred to referral hospital

4. Rate of inter-hospital transfer after receiving initial treatment from peripheral hospital.

Secondary

1. Deaths from poisoning

a. Documented in primary and referral hospital charts and from inter-hospital ambulance transfer records

2. Extent of irreversible acetylcholinesterase inhibition at admission

3. Economic Analysis:
    a. Direct costs of antidote maintenance and patient related costs such as transport would be estimated for their hospital.

In addition to examining overall effectiveness of the intervention in changing specific behaviour, we would examine the persistence of behavioural change and factors that may influence the extent and persistence of any effects

4 Persistence of behavioural change.
    a. The magnitude of effect would be measured over time in each intervention group and in the control to determine if the behavioural change is maintained.

5 Factors that may influence the extent and persistence of behavioral change.
    a. We will (post hoc) examine how variable the effect is and whether factors such as hospital size, staffing, patient load and proximity to referral centers correlate with the extent of behavioural change.

Together with primary secondary outcome data, the basic characteristic of patients will be collected including

- o Demographic Data including gender, age etc.
- o Exposure details – type of poison, amount ingested and ingestion time.

Individual patient level: from secondary hospital

- Admission date and time to the secondary hospital for transferred patients.
- The mode of transfer to the secondary hospital

From hospital:

Antidotes:

- Availability of antidotes as monthly audits– activated charcoal, pralidoxime, atropine.
- Availability of other medication which are essential for emergency care – adrenaline, IV fluids etc.
- Improving of the ordering procedure – regular ordering procedures, records of the ordering books, ordering forms.
- Improving of the storage procedures – storing procedure, using expire date, batch number in storing

Equipments:

- Availability of equipments which are necessary for stabilizing a poisoned patient – Cardiac monitors, ECG machines, laryngoscopes, sucker, ambu bags.
- Details of the regular maintain and storing and ordering accessories for equipments – papers/gel for ECG, battery for laryngoscope.
- Availability of consumable items which are essential for treating and stabilizing poisoned patients.

Medical records/Patient notes:

- Improvement of the data recording after the intervention and the persistent of the change
- Utilization of the newly introduced data recording forms.

Data Collection Tools

Quantitative data:

A special password protected computer based database will be created to enter patient details. Every patient admits to peripheral hospital during the study period will be given an identical study number. Research assistants will enter demographic, exposure, examination, initial management, further management and outcome data

directly to the computers during their hospital visits. These details will be added to the main database in the central office immediately.
Data about antidotes and equipments will be collected from hospital records by research assistants and enter to computer.

Sample size:
We anticipate that a study with one year follow up would allow sufficient recruitment to detect clinically meaningful changes in all our primary outcomes including changes in gastrointestinal decontamination, transfer rates and to detect improved admission cholinesterase activity in organophosphate poisoned patients.

Using an $\alpha=0.05$, $1-\beta=0.8$ for all power estimates and adjusting for clustering assuming an average cluster size of 45 and an intracluster correlation coefficient of 0.05 for 46 hospitals which are included in the study. The value of intracluster correlation coefficient was selected based on data from similar studies conducted in primary hospital set up. However, the estimated number of participants during the planed study period will be sufficient to reach significant sample size even with the highest intracluster correlation of 0.1.

1. **Use of activated charcoal:** An increase from 35% to 50% would require 600 patients in each group. This number of patients should be accrued in approximately 7 months.

2. **Use of forced emesis**: Our conservative estimates are that forced emesis is currently conducted in at least 60% of patients. A clinically significant reduction from 60% to 40% requires 350 patients in each group. This number of patients should be accrued in approximately 4 months.

Persistence of behavioral change: These two key outcomes (1 & 2) will be used to assess changes over time. This will allow us to test whether this degree of clinically significant difference is maintained over the 1 year study period (analyzing each of 4 quarters of post-intervention clinical data compared with the baseline data).

3. **Use of pralidoxime in organophosphate patients:** We estimate that 750 symptomatic patients are transferred to referral hospitals each year. Pralidoxime is currently administered to only about 5% of such patients. To demonstrate an increase from 5% to 25% requires 200 patients in each arm and therefore this could be detected with a year of follow-up data.

Power calculation - Secondary Clinical Outcomes

1. **Deaths from poisoning:** This study is powered to detect a 6% reduction from baseline mortality of 14% for organophosphates (800 in each arm). Previous studies in referral hospitals have shown up to a 30% reduction in mortality by using standard protocols and reducing unnecessary gastric lavage. Further benefit may accrue if patients receive charcoal within one hour and have early administration of the antidote pralidoxime (which is often ineffective after the 3-4 hours median time to admission).

2. **Extent of irreversible acetylcholinesterase inhibition at admission:** It is estimated that 1500 patients with OP poisoning would be transferred over two years. Of these at least half would have severe cholinesterase inhibition (RBC-AChE < 25% of normal – levels below this usually associated with severe cholinergic symptoms). However half of this group would have been likely to have taken dimethoxy or S-alkyl organophosphates which age rapidly. Thus we would expect a good response to be possible in 350-400 patients of whom half will receive pralidoxime. To detect an increase in the % of those with RBC-AChE >25% of normal in cholinesterase function as a response to early pralidoxime from 50% to 60% (i.e. 10% less patients severely poisoned in intervention vs control hospitals) requires 918 patients evenly spread between the three-arms. However, the more appropriate pare wise comparison with Bonferroni adjusted alpha (α=0.0167) requires 537 in each arm.

Statistical analysis:

Data will be cleaned and checked for error while during the collection process as a quality assurance measure and after completion the collection.

As all out outcomes are binary and considered as proportions. Hence χ^2 test for each binary outcome will be applied with adjusted for hospital clusters using logistic regression methods.

Basic characteristics will be checked between groups and within the group.

A logistic mixed model will be fitted to each outcome variable and will include treatment with any potential confounders that imbalance between groups, as fixed effect. A random effect will be included for hospitals to outcome for clustering.

Ethical Considerations:

All the data will be stored in a password protected computers. All the patient records will be given a study number for further references and none of the records will be arranged, used or analyzed based on names or other personal details of the participants.

This study is already approved by committee of Research and Ethics Review, Faculty of Medicine, University of Peradeniya

Reference:

1. Eddlestion M. Patterns and problems of deliberate self-poisoning in the developing world. Q J Med. 2000; **93**: 715-31.

2. Jeyarathnam J. Acute Pesticide Poisoning: A Major Global Health Problem. World Health Statistics Quarterly. 1990; **43**(3): 139-44.

3. Jose M Bertolote AF, Alexander Butchart, Nida Besbelli. Suicide, Suicide attempts and pesticides: a major hidden public health problem. Bull of World health Organisation. 2006; **84**(4).

4. M Eddleston CAA, W P Meyer, G Perera, A M Kularathna, S Attapattu, M H R Sheriff and D A Warrel. Epidemic of self-poisoning with seeds of the yellow oleander tree (Thevetia peruviana) in Nothern Sri Lanka. Tropical Medicine and International Health. 1999; **4**(4): 266-73.

5. Michael Eddleston DG, Ayanthi Karunarathna, Dhammika De Silva, M H Rezvy Sheriff and Nick A Buckley. Epidemiology of intentional self-poisoning in rural Sri Lanka. British Journal of Psychaitry. 2005; **187**: 583-4.

6. Senarathna. How the level of resources and hospital staff attitude affect poisoning patient outcome from primary care hospitals in rural Sri Lanka: University of Newcastle, Australia; 2006.

7. Senarathna L, Adams J, De Silva D, Buckley NA, Dawson AH. Personal and professional challenges in the management of deliberate self-poisoning patients in

rural Sri Lanka: a qualitative study of rural hospital doctors' experiences and perceptions. BMC Public Health. 2008; **8**: 373.

8. W Van Der Hoek FK, K Athukorala and T Wanigasekara. Pesticide Poisoning: A major Health Problem in Sri Lanka. Social Science in Medicine. 1998; **46**(4-5): 495 - 504.

9. Darren Roberts AK, Nick A Buckley, Gamini Manuweera, M H Rezvi Sheriff & Michael Eddleston Influence of pesticide regulation on acute poisoning deaths in Sri Lanka. WHO Bulletine 2003; **81**(10).

10. Eddleston M, Ayanthi Karunaratne, Manjula Weerakoon, Subashini Kumarasinghe, Majula Rajapakshe, M.H. Rezvi Sheriff, Nick A. Buckley, and David Gunnell. "Choice of poison for Intentional self-poisoning in Rural Sri Lanka. Journal of Toxicology: Clinical Toxicology. 2006; **44**(3).

11. Eddleston M, Sudarshan K, Senthilkumaran M, Reginald K, Karalliedde L, Senarathna L, et al. Patterns of hospital transfer for self-poisoned patients in rural Sri Lanka: implications for estimating the incidence of self-poisoning in the developing world. Bull World Health Organ. 2006; **84**(4): 276-82.

12. Witt K, Knudsen E, Ditlevsen S, Hollnagel H. Academic detailing has no effect on prescribing of asthma medication in Danish general practice: a 3-year randomized controlled trial with 12-monthly follow-ups. Fam Pract. 2004; **21**(3): 248-53.

13. Hrisos S, Eccles M, Johnston M, Francis J, Kaner EF, Steen N, et al. An intervention modelling experiment to change GPs' intentions to implement evidence-based practice: using theory-based interventions to promote GP management of upper respiratory tract infection without prescribing antibiotics #2. BMC Health Serv Res. 2008; **8**: 10.

14. Irwin Nazareth NF, Catherine Duggan, James Mason, Andy Haines. Evaluation of a complex intervention for changing professional behaviour: the Evidence Based Out Reach (EBOR) Trial. Journal of Health Services Research & Policy. 2002; **7**(4): 230-8.

15. Kerem Shuval EB, Doron Netzer al Hekselman, Shai Linn, Mayer Brezis, Shmuel Reis Evaluating the impact of an evidence-based medicine educational intervention on primary care doctors’ attitudes, knowledge and clinical behaviour: a controlled trial and before and after study. Journal of Evaluation in Clinical Practice. 2007; **13**: 581-98.

16. Silva SD. The Effectiveness of an Educational Intervention on Behaviour Change Among Health Care Providers on Gastrointestinal Decontamination Practices of Patients with Acute Ingestion of Poison Asia Pacific Association of Medical Toxicology. Chandigrah, India; 2009.

17. Fernando R. Management of poisoning: National Poison Information Centre, National Hospital of Sri Lanka; 2007.
